# A functional genomic screen in *Saccharomyces cerevisiae* reveals divergent mechanisms of resistance to different alkylphosphocholine chemotherapeutic agents

**DOI:** 10.1093/g3journal/jkab233

**Published:** 2021-08-10

**Authors:** Jaquelin M Garcia, Michael J Schwabe, Dennis R Voelker, Wayne R Riekhof

**Affiliations:** 1 School of Biological Sciences, University of Nebraska—Lincoln, Lincoln, NE 68588, USA; 2 Department of Medicine, National Jewish Health, Denver, CO 80206, USA

**Keywords:** Saccharomyces, lipid, phosphatidylcholine, alkylphosphocholine, miltefosine, antifungal, drug resistance, transport

## Abstract

The alkylphosphocholine (APC) class of antineoplastic and antiprotozoal drugs, such as edelfosine and miltefosine, are structural mimics of lyso-phosphatidylcholine (lyso-PC), and are inhibitory to the yeast *Saccharomyces cerevisiae* at low micromolar concentrations. Cytotoxic effects related to inhibition of phospholipid synthesis, induction of an unfolded protein response, inhibition of oxidative phosphorylation, and disruption of lipid rafts have been attributed to members of this drug class, however, the molecular mechanisms of action of these drugs remain incompletely understood. Cytostatic and cytotoxic effects of the APCs exhibit variability with regard to chemical structure, leading to differences in effectiveness against different organisms or cell types. We now report the comprehensive identification of *S. cerevisiae* titratable-essential gene and haploid nonessential gene deletion mutants that are resistant to the APC drug miltefosine (hexadecyl-*O*-phosphocholine). Fifty-eight strains out of ∼5600 tested displayed robust and reproducible resistance to miltefosine. This gene set was heavily enriched in functions associated with vesicular transport steps, especially those involving endocytosis and retrograde transport of endosome derived vesicles to the Golgi or vacuole, suggesting a role for these trafficking pathways in transport of miltefosine to potential sites of action in the endoplasmic reticulum and mitochondrion. In addition, we identified mutants with defects in phosphatidylinositol-4-phosphate synthesis (TetO::*STT4*) and hydrolysis (*sac1*Δ), an oxysterol binding protein homolog (*osh2*Δ), a number of ER-resident proteins, and multiple components of the eisosome. These findings suggest that ER-plasma membrane contact sites and retrograde vesicle transport are involved in the interorganelle transport of lyso-PtdCho and related lyso-phospholipid-like analogs to their intracellular sites of cytotoxic activity.

## Introduction

**Figure 1:**
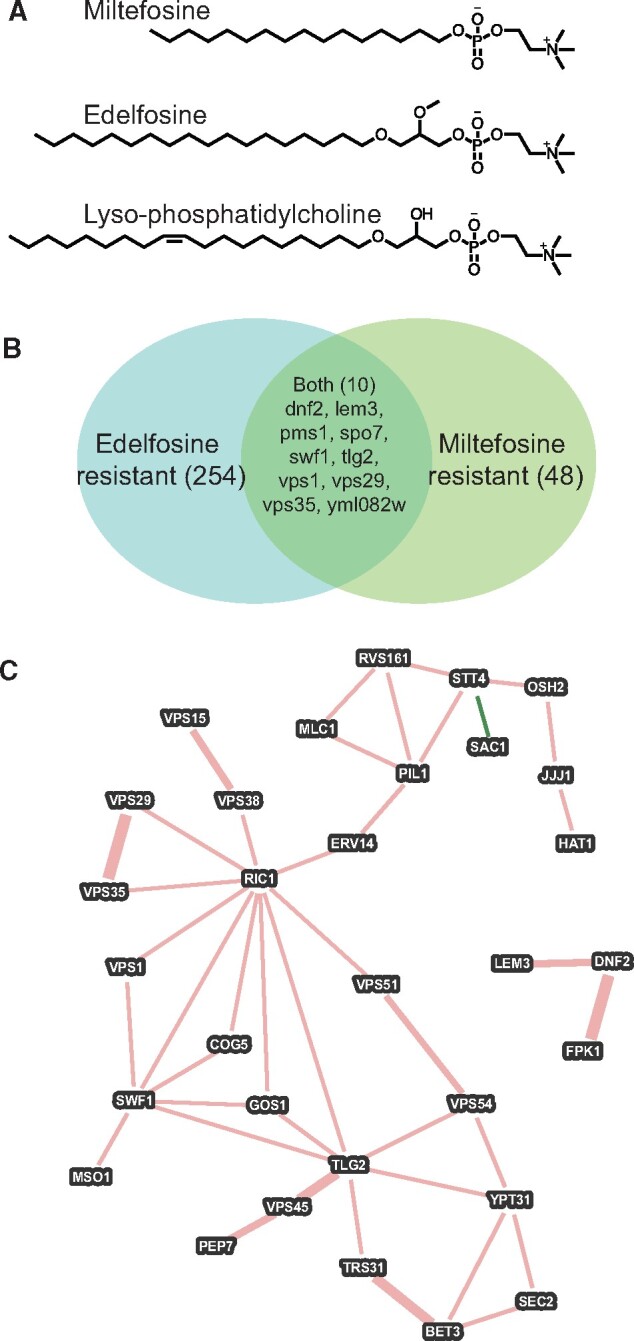
Overlap between previous and current screens for APC resistance. (A) Structures of APC compounds are discussed in the text in comparison with lyso-PC.; (B) Venn diagram illustrating the limited overlap in gene sets between the current study and previous studies on edelfosine resistance.; (C) Physical and genetic interaction network of genes identified as being important for both miltefosine and edelfosine resistance, with additional network neighbors from the miltefosine-only screen described in this work.

The alkylphosphocholine (APC) class of drugs are structural analogs of lyso-phosphatidylcholine (lyso-PC), and have been extensively investigated as antineoplastic agents (Gajate *et al.* 2012; Gajate and Mollinedo 2014). They also act as effective anti-protozoal compounds, with potent activity against *Leishmania* spp. and other apicomplexan parasites (Saraiva *et al.* 2002; Santa-Rita *et al.* 2004; Verma *et al.* 2007; Aichelburg *et al.* 2008; Machado *et al.* 2010). *Leishmania donovani* has been investigated with regard to genetic mechanisms leading to drug resistance, and miltefosine-resistant strains have been identified in which a P_4_-ATPase (lipid flippase) at the plasma membrane is defective, and thus the resistant strain fails to import the toxic compound (Pérez-Victoria *et al.* 2006; Seifert *et al.* 2007; Weingärtner *et al.* 2010). The APC’s are also active against fungal pathogens such as *Candida albicans and Cryptococcus neoformans* (Widmer *et al.* 2006; Vila *et al.* 2015), and the yeast *Saccharomyces cerevisiae* has served as a model for studies on drug resistance and elucidation of the molecular mechanism(s) of action of these drugs (Hanson *et al.* 2003; Cuesta-Marbán *et al.* 2013; Czyz *et al.* 2013). Although the body of work on APC resistance is extensive, particularly on mechanisms of action of edelfosine and miltefosine (Figure 1A), an understanding of the precise mechanism(s) of action of the APC’s remains incomplete, and the intracellular location of targets and mechanisms of action of these drugs appear to vary from compound to compound and between organisms and cell types.

A main mechanism of drug resistance, however, is clearly attributable to the loss of active transport of the APC’s into cells, which occurs via P_4_-family ATPase mediated internalization at the plasma membrane (Hanson *et al.* 2003; Riekhof and Voelker 2009). In the extensively studied *L. donovanii* system, disruption of the miltefosine transporter, LdMT (Pérez-Victoria *et al.* 2006; Seifert *et al.* 2007) or its noncatalytic β-subunit, LdRos3 (Pérez-Victoria *et al.* 2006) leads to drug resistance, similar to loss of the homologous *S. cerevisiae* proteins Dnf2p or Lem3p, respectively, which leads to edelfosine and miltefosine resistance in yeast (Hanson *et al.* 2003). A whole-genome nonessential gene knockout screen for edelfosine resistant and hypersensitive mutants of *S. cerevisiae* was reported in a pair of studies (Cuesta-Marbán *et al.* 2013; Czyz *et al.* 2013). This work showed that edelfosine and miltefosine treatment causes loss of the proton efflux pump Pma1p from detergent-resistant subdomains of the plasma membrane. This leads to an apparent mislocalization of Pma1p in APC treated cells, a corresponding defect in regulation of intracellular pH, and increased sensitivity to low extracellular pH. The edelfosine-resistant gene deletion mutant set these studies identified was enriched in genes encoding proteins with roles in endocytosis and endosomal transport, as well as in intracellular pH regulation.

We now report the comprehensive identification of miltefosine-resistant *S. cerevisiae* haploid deletion and titratable-essential gene strains. While there is overlap with the edelfosine-resistant mutant strains, we identified a substantial number of new mutant strains as miltefosine-resistant, suggesting that the mechanism(s) by which these compounds exert their cytotoxic effects are different, and that the mechanisms of fungal resistance to these drugs is not conserved across this drug class. In addition, we identified an oxysterol-binding protein homolog (Osh2p) as being necessary for miltefosine sensitivity, and suggest that its presence at membrane contact sites is necessary for efficient APC dissemination to sites of action within the cell. We further show that proper Osh2p localization is likely to be dependent on the content and localization of phosphatidylinositol-4-phosphate, as judged by mislocalization of Osh2p-GFP in TetO::*STT4* and *sac1*Δ strains.

## Materials and methods

### Strains and growth conditions

All media components were from Fisher Scientific or Sigma-Aldrich. Routine growth and screening was conducted on YPD (1% w/v yeast extract, 2% w/v peptone, 2% w/v glucose) or YPGro (2% v/v glycerol instead of glucose) solidified with 1.5% w/v agar. Miltefosine was prepared as a 10 mg/ml stock solution in water, filter sterilized, and stored in frozen aliquots until just prior to use. Doxycycline was prepared as a 10 mM stock solution in ethanol and added to media (10 µM final concentration) as needed.

The *MAT*α deletion collection, constructed in parental strain BY4742 (Baker-Brachmann *et al.* 1998), and the tetracycline-repressible essential gene collection, constructed in strain R1158 (Mnaimneh *et al.* 2004), were purchased from Invitrogen. Additional *osh1*Δ, *osh2*Δ, *osh3*Δ, and *osh1*Δ *osh2*Δ *osh3*Δ strains in the SEY6210 background were provided by Tim Levine (University College London.) Initial screens were conducted by thawing 96-well glycerol stock plates, mixing with a stainless steel 96-pin tool (Dan-Kar model MC-96; Fisher Scientific), and dilution into 150 µl of sterile YPD medium in a 96-well plate. Approximately 3 µl of culture is transferred by each pin under these conditions, giving a ∼50 fold dilution in the recipient plate. The diluted cultures were then pinned to solid YPD medium with or without 4 µg/ml (9.8 µM) miltefosine (Avanti Polar Lipids, Alabaster, AL, USA), incubated at 30 °C for up to 7 days, and visually monitored daily for growth of miltefosine-resistant patches. Cultures from the essential doxycycline-repressible (“Tet-off”) promoter collection (Mnaimneh *et al.* 2004) were screened similarly, except that an additional series of plates were used for the screen, which included 10 µg/ml doxycycline to affect repression of the essential library gene, as previously described (Wishart *et al.* 2005).

Miltefosine-resistant strains were identified and subcultured for further studies. Resistant strains were colony-purified from patches on the uninhibited miltefosine-free YPD replica plates, and single colonies were picked and grown to saturation in YPD. A 10-fold dilution series was prepared in 96-well plates and pinned to YPD (4 µg/ml miltefosine) or YPGro (1 µg or 4 µg/ml miltefosine) and growth assessed after 48 or 96 hours, respectively. Strains that showed reproducible miltefosine resistance were thus identified, and categorized based on degree of sensitivity with glucose or glycerol as carbon sources.

### Bioinformatic analyses

Primary and secondary screening led to the identification of 58 genes which, when deleted or repressed, led to a reproducible miltefosine-resistant growth phenotype. Gene ontology (GO) analysis was performed with YeastMine tools available at the Saccharomyces Genome Database website (www.yeastgenome.org), and gene set analysis was conducted with YeastNet version 3.0 at www.inetbio.org/yeastnet (Kim *et al.* 2014).

### Microscopy

Strain BY4742 and the isogenic *sac1Δ*::*KanMX* deletion strain were transformed to uracil prototrohy with plasmid pTS312, a *URA3 CEN* plasmid expressing a C-terminal GFP fusion of Osh2p, which was a gift of Christopher Beh (Simon Fraser University.) Cultures were grown overnight in SC -Ura and imaged on an Evos FL inverted microscope with GFP fluorescence cube.

## Results and discussion

### Screening for miltefosine resistant strains identifies a network of 58 highly connected genes enriched in membrane and trafficking functions

**Figure 2:**
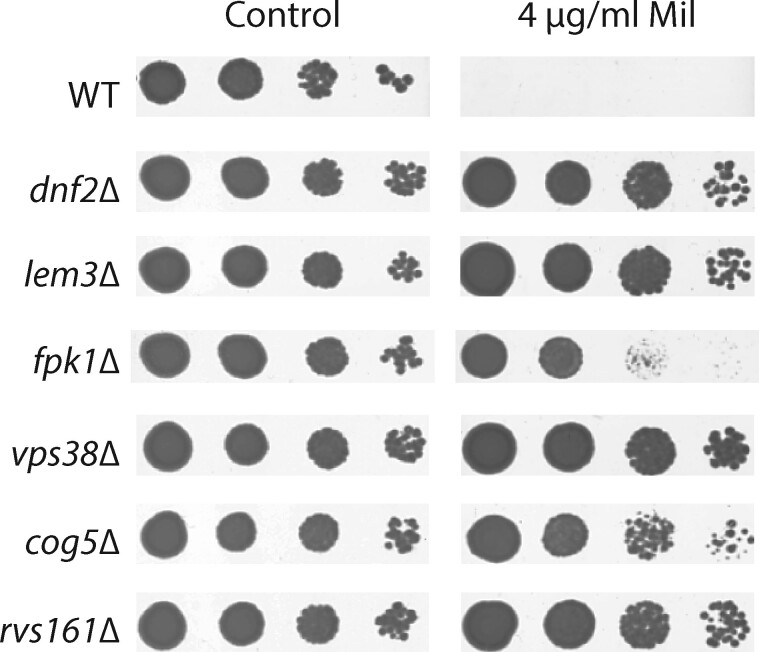
Miltefosine-resistance phenotypes. Primary screening led to the identification of strains with a range of resistance phenotypes. Strains were grown overnight in YPD and a fivefold serial dilution series was prepared and pinned to solid YPD agar with (4 µg/ml Mil) or without (control) miltefosine. Plates were photographed after 3 days. The densest spots correspond to the 1:25 dilution, and the lightest corresponds to 1:3125 dilution. A range of phenotypes from weakly (*fpk1Δ*) to modestly (*cog5*Δ) to strongly (*dnf2*Δ and *vps38*Δ) resistant are noted.

Table 1 provides a list of the genes that were identified as showing reproducible resistance to 4 µg/ml miltefosine on YPD, and that rescreened as positive after colony purification of original stocks and assessment with a fivefold serial dilution spot-test. Relevant molecular structures are shown in Figure 1A. Figure 2 shows a typical re-screening result for a subset of 6 mutants from the haploid MATα BY4742 based mutant collection. These initial results demonstrated that the miltefosine-resistance phenotype is tractable in the context of a genome-wide deletion screen conducted on solid medium. Figure 5 shows a result typical of the essential-gene Tet-off screening phenotype (TetO::*STT4*), in which we selected strains that showed robust growth in the presence of miltefosine when doxycycline was present (gene repressed), and weak or absent growth in its absence (gene expressed normally).

**Table 1:** List of genes identified in the miltefosine-resistance screen

Gene ID	Name	Previous ID	Description
YAL009W	SPO7	Yes	Subunit of Nem1p-Spo7p phosphatase holoenzyme
YAL039C	CYC3	No	Cytochrome c heme lyase
YBR097W	VPS15	No	Protein kinase involved in vacuolar protein sorting
YBR184W		No	Putative protein of unknown function
YCR009C	RVS161	No	Amphiphysin-like lipid raft protein
YCR067C	SED4	No	ER-protein that stimulates Sar1p GTPase activity
YDL019C	OSH2	No	Oxysterol-binding protein homolog family member
YDR027C	VPS54	No	Golgi-associated retrograde protein complex
YDR028C	REG1	No	Subunit of type 1 protein phosphatase Glc7p
YDR093W	DNF2	Yes	Aminophospholipid translocase (flippase)
YDR095C		No	Dubious ORF overlapping *DNF2*
YDR097C	MSH6	No	Required for mismatch repair in mitosis and meiosis
YDR126W	SWF1	Yes	Palmitoyltransferase of SNARE proteins
YDR323C	PEP7	No	Vesicle-mediated vacuolar protein sorting
YDR472W	TRS31	No	Transport protein particle (TRAPP) I-III
YER031C	YPT31	No	Rab family GTPase
YFL047W	RGD2	No	GTPase-activating protein for Cdc42p and Rho5p
YGL012W	ERG4	No	C-24(28) sterol reductase
YGL054C	ERV14	No	COPII-coated vesicle protein
YGL095C	VPS45	No	Protein of the Sec1p/Munc-18 family
YGL106W	MLC1	No	Essential light chain for Myo1p
YGL158W	RCK1	No	Protein kinase involved in oxidative stress response
YGR086C	PIL1	No	Eisosome core component
YGR141W	VPS62	No	Vacuolar protein sorting (VPS) protein
YHL028W	WSC4	No	Endoplasmic reticulum (ER) membrane protein
YHL031C	GOS1	No	v-SNARE protein involved in Golgi transport
YHR012W	VPS29	Yes	Subunit of retromer complex
YHR108W	GGA2	No	Regulates Arf1p, Arf2p to facilitate Golgi trafficking
YJL154C	VPS35	Yes	Endosomal subunit of retromer complex
YKL212W	SAC1	No	PtdIns-4-phosphate phosphatase
YKR001C	VPS1	Yes	Dynamin-like GTPase required for vacuolar sorting
YKR019C	IRS4	No	EH domain-containing protein
YKR020W	VPS51	No	Golgi-associated retrograde protein complex
YKR068C	BET3	No	Transport protein particle (TRAPP) complexes I-III
YLR039C	RIC1	No	Retrograde transport to the cis-Golgi network
YLR082C	SRL2	No	Protein of unknown function
YLR093C	NYV1	No	v-SNARE component of vacuolar membrane
YLR305C	STT4	No	Phosphatidylinositol-4-kinase
YLR360W	VPS38	No	Vps34p phosphatidylinositol-3-kinase complex
YML052W	SUR7	No	Plasma membrane component of eisosomes
YML082W		Yes	Protein of unknown function
YMR032W	HOF1	No	Regulates actin cytoskeleton organization
YNL051W	COG5	No	Conserved oligomeric Golgi complex subunit
YNL058C		No	Putative protein of unknown function
YNL082W	PMS1	Yes	ATP-binding protein required for mismatch repair
YNL227C	JJJ1	No	Stimulates the ATPase activity of Ssa1p
YNL272C	SEC2	No	Guanyl-exchange factor for small G-protein Sec4p
YNL323W	LEM3	Yes	Beta-subunit of Lem3p flippase
YNR047W	FPK1	No	Ser/Thr protein kinase activating Dnf2p
YNR049C	MSO1	No	Lipid-interacting protein in SNARE assembly
YOL018C	TLG2	Yes	Syntaxin-like t-SNARE
YOL107W		No	Putative protein of unknown function
YOR311C	DGK1	No	Diacylglycerol kinase
YPL001W	HAT1	No	Hat1p-Hat2p histone acetyltransferase complex
YPL028W	ERG10	No	Acetyl-CoA C-acetyltransferase
YPR089W		No	Protein of unknown function
YPR117W		No	Putative protein of unknown function
YPR151C	SUE1	No	Degradation of unstable forms of cytochrome c

Genes are listed in order of chromosomal position as noted from the standard SGD identifier, with gene name (if available), assessment of whether the mutant had previously been identified as resistant to edelfosine or miltefosine, and a description of the gene product.

**Figure 3:**
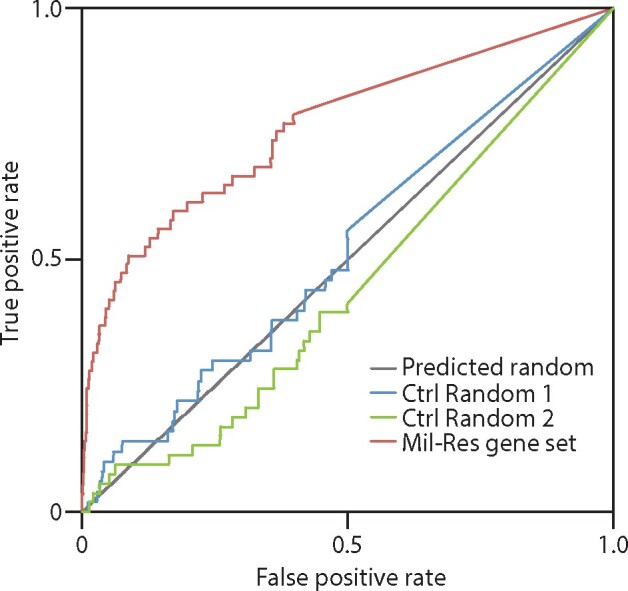
Receiver-operating characteristic curve analysis. The gene set of reproducibly miltefosine resistant mutants was analyzed with gene-set enrichment analysis tools from the YeastNet v3 package at https://www.inetbio.org/yeastnet/as described in the text. Two sets of randomly selected genes were analyzed for comparison, and the miltefosine-resistant gene set was found to identify genes that were significantly connected to each other via protein: protein interactions, genetic epistasis, shared domain architectures, and co-citation indices with an *E*-value of less than 1 × 10^−10^.

When screening mutant libraries for strains with alterations in a phenotype of interest, it is useful to assess whether the screen has identified mutants in a subset of genes with related functions, or whether the screen has identified a set of diverse genes with little in common with regard to function or localization. We approached this question by assessing the connectivity of the identified gene set with regard to systems-based epistasis screens, protein–protein interaction studies, and other measures, such as shared protein domain architectures and co-citation indices. We also analyzed the enrichment of GO terms associated with the gene set. The method of assessment of connectivity makes use of receiver operating characteristic (ROC) curve analysis (McGary *et al.* 2007), in which the area under the ROC curve is assessed in relation to that expected for a randomly chosen subset of genes. The results of ROC analysis for our set of 58 genes are given in Figure 3. This approach makes use of the YeastNet v3 database (Kim *et al.* 2014) to assess whether a set of genes are more connected to each other than they are to a random subset of the genome. This approach led us to discover that miltefosine resistance is a powerful selection for mutants in a subset of genes that are highly connected via genetic interactions, protein: protein interactions, and co-citation indices with one another, with a *P*-value of 8.3 × 10^−11^. This means that that the chances of identifying a random set of genes with this level of connectivity are on the order of 1 in 10^−10^. As a control, we selected two random pools of 58 genes each and analyzed their connectivity by ROC analysis for the miltefosine-resistance gene set. As expected, these subsets of genes displayed no significant numbers of connections beyond that which would be expected from random chance.

A second method of gene set analysis was performed by assessing the enrichment of GO terms as a measure of relatedness, and allowed us to identify molecular components and processes that are significantly over-represented in the miltefosine-resistant mutant collection. Table 2 provides a list of GO terms that are over-represented in the miltefosine-resistant gene set. As might be expected for a set of mutants with resistance to a membrane-perturbing agent, GO components and processes associated with membrane assembly, protein trafficking, and transport through the endomembrane system are highly enriched in this analysis.

**Table 2:** Gene-ontology term enrichment

GO identifier	GO component (C) or process (P)	Total for term	Identified	Fold enrichment	*P*-value
GO: 0005794	C: Golgi apparatus	214	17	8.2	6.92E-12
GO: 0000139	C: Golgi membrane	121	10	8.5	1.77E-07
GO: 0031201	C: SNARE complex	25	5	20.7	3.26E-06
GO: 0005768	C: endosome	114	8	7.2	1.16E-05
GO: 0016020	C: membrane	1718	31	1.9	5.54E-05
GO: 0010008	C: endosome membrane	59	5	8.8	0.0002392
GO: 0000938	C: GARP complex	4	2	51.6	0.0005458
GO: 0034272	C: phosphatidylinositol 3-kinase complex II	4	2	51.6	0.0005458
GO: 0030906	C: retromer complex, inner shell	4	2	51.6	0.0005458
GO: 0030904	C: retromer complex	5	2	41.3	0.0009041
GO: 0006810	P: transport	793	22	2.9	1.74E-06
GO: 0015031	P: protein transport	389	17	4.5	7.43E-08
GO: 0016192	P: vesicle-mediated transport	140	10	7.4	6.98E-07
GO: 0006897	P: endocytosis	88	8	9.4	1.64E-06
GO: 0006896	P: Golgi to vacuole transport	24	5	21.5	2.63E-06
GO: 0006623	P: protein targeting to vacuole	48	5	10.8	8.87E-05
GO: 0042147	P: retrograde transport, endosome to Golgi	19	5	27.2	7.46E-07
GO: 0006914	P: autophagy	51	4	8.1	0.001407
GO: 0032258	P: CVT pathway	38	4	10.9	0.0004564
GO: 0006906	P: vesicle fusion	20	4	20.7	3.41E-05
GO: 0006895	P: Golgi to endosome transport	14	3	22.1	0.0002905
GO: 0006869	P: lipid transport	21	3	14.8	0.001012
GO: 0045053	P: protein retention in Golgi apparatus	11	3	28.2	0.0001344
GO: 0000011	P: vacuole inheritance	18	3	17.2	0.0006335
GO: 0006904	P: vesicle docking involved in exocytosis	10	3	31.0	9.84E-05
GO: 0048017	P: inositol lipid-mediated signaling	5	2	41.3	0.0009041
GO: 0060988	P: lipid tube assembly	3	2	68.9	0.0002746

Genes identified in the mutant screen were analyzed with GO-based gene-set enrichment analysis tools available at www.yeastgenome.org. *P*-values were calculated based on the hypergeometric test, and GO terms with *P* < 0.005 are included in the table.

### The miltefosine resistant gene deletion set shows little overlap with a previously described edelfosine resistance screen

As noted in Figure 1, approximately 264 deletion mutant strains were previously identified as showing some degree of edelfosine resistance (Cuesta-Marbán *et al.* 2013; Czyz *et al.* 2013). Our current study identified 58 mutants showing resistance to miltefosine, and of those, only 10 mutants were shared between the edelfosine and miltefosine resistance sets, although this may be a slight underestimate given that the edelfosine resistance screen did not encompass the essential gene titratable-promoter collection. Figure 1C shows a composite genetic and physical interaction map of edelfosine-miltefosine resistant shared genes and their directly interacting partners in the miltefosine resistance set, and identifies the t-SNARE TLG2 as a key hub in the interaction diagram for the shared edelfosine-miltefosine resistant gene set.

A *tlg2*Δ strain was identified along with *dnf2*Δ, *lem3*Δ, and other components of endosome-plasma membrane recycling in a screen for strains resistant to the lantibiotic peptide Ro 09-0198 (Takagi *et al.* 2012)*.* This work also showed that an EGFP-tagged Dnf2p reporter was mislocalized in the *tlg2*Δ background, suggesting that the Ro-0198 sensitive phenotype of *tlg2*Δ is due to mislocalization of Dnf2p from the plasma membrane to endosomes. Disruption or mislocalization of this lipid flippase would thus result in the accumulation of phosphatidylethanolamine on the outer leaflet of the plasma membrane, which is the ligand for this cytolytic peptide. Taken together with our results, this suggests that localization and proper function of the Dnf2p-Lem3p complex is dependent on Tlg2p and a small cadre of proteins to which it is functionally linked, and that the genes in the intersection of the Venn diagram of Figure 1B and in the interaction diagram of Figure 1C encompass the core components necessary for flippase function, activity, and localization at the plasma membrane. We thus propose that while the core components of lysophospholipid uptake (the flippase core components and factors regulating its proper localization and function) are broadly conserved as determinants of APC sensitivity, individual members of this drug class exert their cytotoxic effects by interacting with multiple and variable intracellular targets after their import via the flippase.

### Disruption of phosphatidylinositol-4-phosphate homeostasis and the oxysterol-binding protein homolog Osh2p alters miltefosine sensitivity

The proteins disrupted in a subset of functionally related miltefosine-resistant mutants (*osh2*Δ, *sac1*Δ, and TetO::*STT4*) are involved in the function and localization of the oxysterol binding protein homolog Osh2p. Osh2p contains an oxysterol-binding domain at its C-terminus as well as several protein–protein and protein: lipid interaction motifs at its N-terminus. These interaction motifs include Anykyrin-repeats which are likely to interact with other, currently unidentified, proteins (Beh *et al.* 2001), a Pleckstrin-homology (PH) domain that interacts with PtdIns-4-P (Roy and Levine 2004), and a “two phenylalanines in an acidic tract” (FFAT) motif which interacts with the ER resident proteins Scs2p and Scs22p (Loewen *et al.* 2003; Kaiser *et al.* 2005; Loewen and Levine 2005).

**Figure 4:**
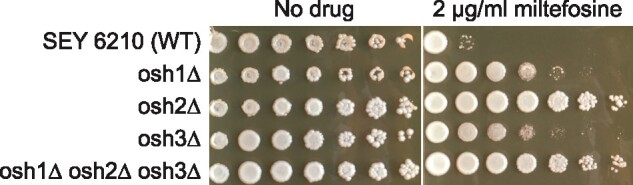
Oxysterol binding protein homologs are necessary for wild-type miltefosine sensitivity. An osh2Δ strain was identified in the primary screen conducted in the BY4742 background. To confirm the role of Osh2p and its orthologs Osh1p and Osh3p and determine whether there were background-specific differences in sensitivity, we assessed the growth of strain SEY6210 and isogenic *osh1*Δ, *osh2*Δ, *osh2*Δ, and the triple mutant. Overnight cultures were subjected to fivefold serial dilution and pinned to solid media as described in the text.

**Figure 5:**
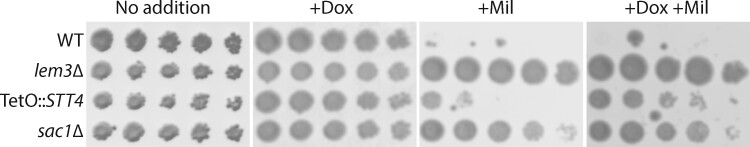
The phosphatidylinositol-4-kinase/phosphatase cycle is a determinant of miltefosine sensitivity. The essential gene *STT4*, encoding the major PtdIns-4-kinase isoform in yeast, was identified in the essential-titratable gene collection. Inhibition of *STT4* expression by including doxycycline in the growth media (10 µM) led to mitefosine resistance (4 µg/ml) The PtdIns-4-P phosphatase Sac1p, was also identified, and a *sac1*Δ strain was included in this series, and *lem3*Δ was included as a previously characterized miltefosine resistant positive control. Fivefold serial dilutions were prepared and pinned to solid media as described in the text.

**Figure 6:**
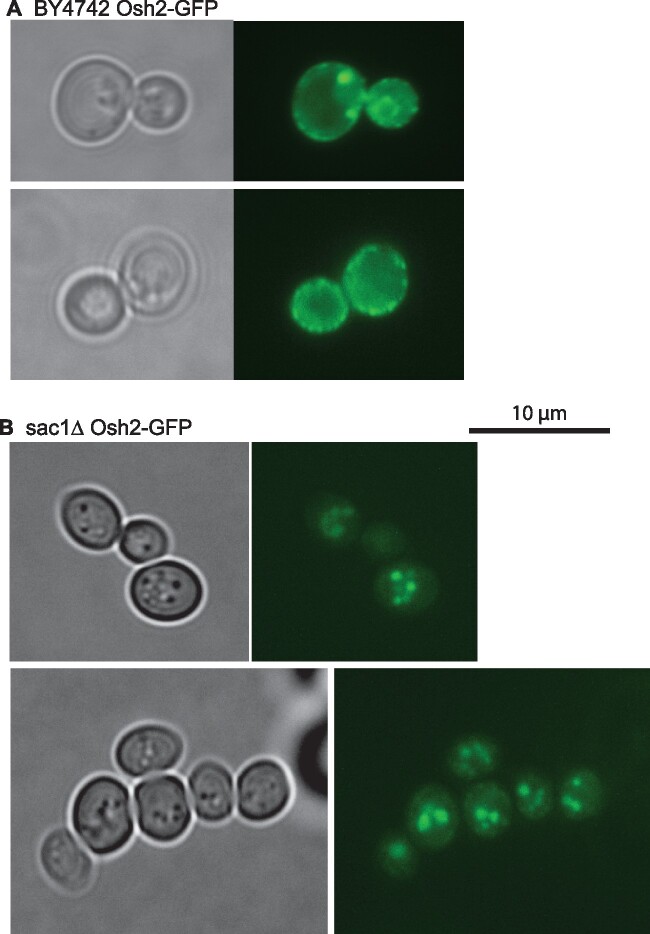
Osh2p is mislocalized in a sac1Δ mutant background. Osh2-GFP expressed from a *URA3* CEN plasmid under its native promoter was introduced into the BY4742 wild-type and isogenic *sac1*Δ mutant strains. Strains were grown in SC -Ura media overnight and images were acquired on an Evos-*Fl* inverted microscope with a GFP light cube using a 100× oil-immersion objective.

The phosphatidylinositol (PtdIns) 4-kinase Stt4p (Yoshida *et al.* 1994; Cutler *et al.* 1997) generates PtdIns-4-P at the plasma membrane (Foti *et al.* 2001; Baird *et al.* 2008), and is involved in actin polymerization and endocytosis, as well as in transport of PtdSer from the ER to the site of Psd2p (Trotter *et al.* 1998). The pool of PtdIns-4-P generated by Stt4p is degraded by the phosphoinositide phosphatase Sac1p (Foti *et al.* 2001), which has additional functions in lipid trafficking and metabolism (Guo *et al.* 1999; Rivas *et al.* 1999; Hughes *et al.* 2000; Tahirovic *et al.* 2005; Riekhof *et al.* 2014; Tani and Kuge 2014). The strains *sac1*Δ, *osh2*Δ, and TetO::*STT4* were all identified as miltefosine resistant (Figures 4 and 5), suggesting that the PtdIns-4-P cycle governed by Stt4p and Sac1p might be involved in the proper localization of Osh2p, and that mislocalization of Osh2p might lead to a defect in miltefosine uptake and distribution to intracellular targets. This idea was tested by expressing a GFP-tagged form of Osh2p in a wild-type and *sac1*Δ background. As shown in Figure 6, Osh2p in the wild type is localized in a punctate pattern at the cell periphery, while in the *sac1*Δ mutant Osh2p is localized to intracellular structures and absent from the cell periphery.

### Summary

Previous studies in *S. cerevisiae* (Cuesta-Marbán *et al.* 2013; Czyz *et al.* 2013) have identified a subset of genes that, when deleted, confer resistance to the APC analog edelfosine. While members of this drug class share superficial similarities to lysophosphatidylcholine in their structures, it has remained an open question as to the degree of overlap in their mechanisms of action. Our current work identifies the plasma membrane flippase Dnf2p and factors required for its correct localization and function as being shared between edelfosine, miltefosine, and likely other members of this drug class with regard to the specific and active transport of the compounds into the cell. However, unlike the edelfosine studies, we did not identify factors that regulate intracellular pH or alter the function of the plasma membrane proton pump Pma1p as having a role in altered miltefosine sensitivity. We did however identify a new subset of genes as determinants of wild-type miltefosine sensitivity, and chose to assess the roles of the PtdIns-4-P cycle and Osh2 localization in more detail. One interpretation of this data is that after the initial “flip” across the plasma membrane by Dnf2p/Lem3, miltefosine is transported to one or more sites of cytotoxic activity by nonvesicular transport routes that are dependent on the proper localization of the oxysterol-binding protein homolog Osh2p. Future work on this and other clusters of genes identified in this screen will provide additional insight into the mechanism(s) of action of miltefosine and other members of the APC drug class.

## Data availability

All data and materials, including strains and plasmids, described in this work are freely available upon request.

## References

[jkab233-B1] Aichelburg AC , Walochnik J, Assadian O, Prosch H, Steuer A, et al 2008. Successful treatment of disseminated Acanthamoeba sp. infection with miltefosine. Emerg Infect Dis. 14:1743–1746. 18976559 10.3201/eid1411.070854 PMC2630722

[jkab233-B2] Baird D , Stefan C, Audhya A, Weys S, Emr SD. 2008. Assembly of the PtdIns 4-kinase Stt4 complex at the plasma membrane requires Ypp1 and Efr3. J Cell Biol. 183:1061–1074. 19075114 10.1083/jcb.200804003 PMC2600738

[jkab233-B3] Baker-Brachmann C , Davies A, Cost GJ, Caputo E, Li J, et al 1998. Designer deletion strains derived from *Saccharomyces cerevisiae*. Yeast. 14:115–132. 9483801 10.1002/(SICI)1097-0061(19980130)14:2<115::AID-YEA204>3.0.CO;2-2

[jkab233-B4] Beh CT , Cool L, Phillips J, Rine J, Adams A, et al 2001. Overlapping functions of the yeast oxysterol-binding protein homologues. Genetics. 157:1117–1140. 11238399 10.1093/genetics/157.3.1117 PMC1461579

[jkab233-B5] Cuesta-Marbán Á , Botet J, Czyz O, Cacharro LM, Gajate C, et al 2013. Drug uptake, lipid rafts, and vesicle trafficking modulate resistance to an anticancer lysophosphatidylcholine analogue in yeast. J Biol Chem. 288:8405–8418. 23335509 10.1074/jbc.M112.425769 PMC3605657

[jkab233-B6] Cutler NS , Heitman J, Cardenas ME. 1997. STT4 is an essential phosphatidylinositol 4-kinase that is a target of wortmannin in *Saccharomyces cerevisiae*. J Biol Chem. 272:27671–27677. 9346907 10.1074/jbc.272.44.27671

[jkab233-B7] Czyz O , Bitew T, Cuesta-Marbán A, McMaster CR, Mollinedo F, et al 2013. Alteration of plasma membrane organization by an anticancer lysophosphatidylcholine analogue induces intracellular acidification and internalization of plasma membrane transporters in yeast. J Biol Chem. 288:8419–8432. 23344949 10.1074/jbc.M112.425744 PMC3605658

[jkab233-B8] Foti M , Audhya A, Emr SD. 2001. Sac1 lipid phosphatase and Stt4 phosphatidylinositol 4-kinase regulate a pool of phosphatidylinositol 4-phosphate that functions in the control of the actin cytoskeleton and vacuole morphology. Mol Biol Cell. 12:2396–2411. 11514624 10.1091/mbc.12.8.2396 PMC58602

[jkab233-B9] Gajate C , Matos-Da-Silva M, Dakir ELH, Fonteriz RI, Alvarez J, et al 2012. Antitumor alkyl-lysophospholipid analog edelfosine induces apoptosis in pancreatic cancer by targeting endoplasmic reticulum. Oncogene. 31:2627–2639. 22056873 10.1038/onc.2011.446

[jkab233-B10] Gajate C , Mollinedo F. 2014. Lipid rafts, endoplasmic reticulum and mitochondria in the antitumor action of the alkylphospholipid analog edelfosine. Anticancer Agents Med Chem. 14:509–527. 24628241 10.2174/1871520614666140309222259

[jkab233-B11] Guo S , Stolz LE, Lemrow SM, York JD. 1999. SAC1-like domains of yeast SAC1, INP52, and INP53 and of human synaptojanin encode polyphosphoinositide phosphatases. J Biol Chem. 274:12990–12995. 10224048 10.1074/jbc.274.19.12990

[jkab233-B12] Hanson PK , Malone L, Birchmore JL, Nichols JW. 2003. Lem3p is essential for the uptake and potency of alkylphosphocholine drugs, edelfosine and miltefosine. J Biol Chem. 278:36041–36050. 12842877 10.1074/jbc.M305263200

[jkab233-B13] Hughes WE , Woscholski R, Cooke FT, Patrick RS, Dove SK, et al 2000. SAC1 encodes a regulated lipid phosphoinositide phosphatase, defects in which can be suppressed by the homologous Inp52p and Inp53p phosphatases. J Biol Chem. 275:801–808. 10625610 10.1074/jbc.275.2.801

[jkab233-B14] Kaiser SE , Brickner JH, Reilein AR, Fenn TD, Walter P, et al 2005. Structural basis of FFAT motif-mediated ER targeting. Structure. 13:1035–1045. 16004875 10.1016/j.str.2005.04.010

[jkab233-B15] Kim H , Shin J, Kim E, Kim H, Hwang S, et al 2014. YeastNet v3: a public database of data-specific and integrated functional gene networks for *Saccharomyces cerevisiae*. Nucleic Acids Res. 42:D731–D736. 24165882 10.1093/nar/gkt981 PMC3965021

[jkab233-B16] Loewen C , Levine T. 2005. A highly conserved binding site in VAP for the FFAT motif of lipid binding proteins. J Biol Chem. 280:14097–14104. 15668246 10.1074/jbc.M500147200

[jkab233-B17] Loewen CJR , Roy A, Levine TP. 2003. A conserved ER targeting motif in three families of lipid binding proteins and in Opi1p binds VAP. EMBO J. 22:2025–2035. 12727870 10.1093/emboj/cdg201 PMC156073

[jkab233-B18] Machado PR , Ampuero J, Guimaraes LH, Villasboas L, Rocha AT, et al 2010. Miltefosine in the treatment of cutaneous leishmaniasis caused by *Leishmania braziliensis* in Brazil: a randomized and controlled trial. PLoS Negl Trop Dis. 4:1–6. 10.1371/journal.pntd.0000912 PMC3006132 21200420

[jkab233-B19] McGary KL , Lee I, Marcotte EM. 2007. Broad network-based predictability of *Saccharomyces cerevisiae* gene loss-of-function phenotypes. Genome Biol. 8:R258. 18053250 10.1186/gb-2007-8-12-r258 PMC2246260

[jkab233-B20] Mnaimneh S , Davierwala AP, Haynes J, Moffat J, Peng WT, et al 2004. Exploration of essential gene functions via titratable promoter alleles. Cell. 118:31–44. 15242642 10.1016/j.cell.2004.06.013

[jkab233-B21] Pérez-Victoria FJ , Sánchez-Cañete MP, Castanys S, Gamarro F. 2006. Phospholipid translocation and miltefosine potency require both *L. donovani* miltefosine transporter and the new protein LdRos3 in Leishmania parasites. J Biol Chem. 281:23766–23775. 16785229 10.1074/jbc.M605214200

[jkab233-B22] Riekhof WR , Voelker DR. 2009. The yeast plasma membrane P4-ATPases are major transporters for lysophospholipids. Biochim Biophys Acta. 1791:620–627. 19268715 10.1016/j.bbalip.2009.02.013

[jkab233-B23] Riekhof WR , Wu W-I, Jones JL, Nikrad M, Chan MM, et al 2014. An assembly of proteins and lipid domains regulates transport of phosphatidylserine to phosphatidylserine decarboxylase 2 in *Saccharomyces cerevisiae*. J Biol Chem. 289:5809–5819. 24366873 10.1074/jbc.M113.518217 PMC3937652

[jkab233-B24] Rivas MP , Kearns BG, Xie Z, Guo S, Sekar MC, et al 1999. Pleiotropic alterations in lipid metabolism in yeast *sac1* mutants: relationship to “bypass Sec14p” and inositol auxotrophy. Mol Biol Cell. 10:2235–2250. 10397762 10.1091/mbc.10.7.2235 PMC25439

[jkab233-B25] Roy A , Levine TP. 2004. Multiple pools of phosphatidylinositol 4-phosphate detected using the pleckstrin homology domain of Osh2p. J Biol Chem. 279:44683–44689. 15271978 10.1074/jbc.M401583200

[jkab233-B26] Santa-Rita RM , Henriques-Pons A, Barbosa HS, de Castro SL. 2004. Effect of the lysophospholipid analogues edelfosine, ilmofosine and miltefosine against Leishmania amazonensis. J Antimicrob Chemother. 54:704–710. 15329361 10.1093/jac/dkh380

[jkab233-B27] Saraiva VB , Gibaldi D, Previato JO, Mendonça-Previato L, Bozza MT, et al 2002. Proinflammatory and cytotoxic effects of hexadecylphosphocholine (miltefosine) against drug-resistant strains of *Trypanosoma cruzi*. Antimicrob Agents Chemother. 46:3472–3477. 12384352 10.1128/AAC.46.11.3472-3477.2002 PMC128733

[jkab233-B28] Seifert K , Pérez-Victoria FJ, Stettler M, Sánchez-Cañete MP, Castanys S, et al 2007. Inactivation of the miltefosine transporter, LdMT, causes miltefosine resistance that is conferred to the amastigote stage of *Leishmania donovani* and persists *in vivo*. Int J Antimicrob Agents. 30:229–235. 17628445 10.1016/j.ijantimicag.2007.05.007

[jkab233-B29] Tahirovic S , Schorr M, Mayinger P. 2005. Regulation of intracellular phosphatidylinositol-4-phosphate by the Sac1 lipid phosphatase. Traffic. 6:116–130. 15634212 10.1111/j.1600-0854.2004.00255.x

[jkab233-B30] Takagi K , Iwamoto K, Kobayashi S, Horiuchi H, Fukuda R, et al 2012. Involvement of Golgi-associated retrograde protein complex in the recycling of the putative Dnf aminophospholipid flippases in yeast. Biochem Biophys Res Commun. 417:490–494. 22177957 10.1016/j.bbrc.2011.11.147

[jkab233-B31] Tani M , Kuge O. 2014. Involvement of Sac1 phosphoinositide phosphatase in the metabolism of phosphatidylserine in the yeast *Saccharomyces cerevisiae*. Yeast. 31:145–158. 24578286 10.1002/yea.3004

[jkab233-B32] Trotter PJ , Wu WI, Pedretti J, Yates R, Voelker DR. 1998. A genetic screen for aminophospholipid transport mutants identifies the phosphatidylinositol 4-kinase, Stt4p, as an essential component in phosphatidylserine metabolism. J Biol Chem. 273:13189–13196. 9582361 10.1074/jbc.273.21.13189

[jkab233-B33] Verma NK , Singh G, Dey CS. 2007. Miltefosine induces apoptosis in arsenite-resistant *Leishmania donovani* promastigotes through mitochondrial dysfunction. Exp Parasitol. 116:1–13. 17161839 10.1016/j.exppara.2006.10.007

[jkab233-B34] Vila TVM , Chaturvedi AK, Rozental S, Lopez-Ribot JL. 2015. Characterization of the *in vitro* activity of Miltefosine against *Candida albicans* under planktonic and biofilm growing conditions and *in vivo* efficacy in the murine model of oral candidiasis. Antimicrob Agents Chemother. 59:7611–7620. 26416861 10.1128/AAC.01890-15 PMC4649157

[jkab233-B35] Weingärtner A , Drobot B, Herrmann A, Sánchez-Cañete MP, Gamarro F, et al 2010. Disruption of the lipid-transporting ldmt-ldros3 complex in *Leishmania donovani* affects membrane lipid asymmetry but not host cell invasion. PLoS One. 5:e12443. 20865154 10.1371/journal.pone.0012443 PMC2928753

[jkab233-B36] Widmer F , Wright LC, Obando D, Handke R, Ganendren R, et al 2006. Hexadecylphosphocholine (miltefosine) has broad-spectrum fungicidal activity and is efficacious in a mouse model of cryptococcosis. Antimicrob Agents Chemother. 50:414–421. 16436691 10.1128/AAC.50.2.414-421.2006 PMC1366877

[jkab233-B37] Wishart JA , Hayes A, Wardleworth L, Zhang N, Oliver SG. 2005. Doxycycline, the drug used to control the tet-regulatable promoter system, has no effect on global gene expression in *Saccharomyces cerevisiae*. Yeast. 22:565–569. 15942933 10.1002/yea.1225

[jkab233-B38] Yoshida S , Ohya Y, Goebl M, Nakano A, Anraku Y. 1994. A novel gene, STT4, encodes a phosphatidylinositol 4-kinase in the PKC1 protein kinase pathway of *Saccharomyces cerevisiae*. J Biol Chem. 269:1166–1171. 8288577

